# Intraperitoneal administration of human “Neo-Islets”, 3-D organoids of mesenchymal stromal and pancreatic islet cells, normalizes blood glucose levels in streptozotocin-diabetic NOD/SCID mice: Significance for clinical trials

**DOI:** 10.1371/journal.pone.0259043

**Published:** 2021-10-28

**Authors:** Christof Westenfelder, Zhuma Hu, Ping Zhang, Anna Gooch

**Affiliations:** 1 Department of Medicine, Division of Nephrology, University of Utah, Salt Lake City, UT, United States of America; 2 SymbioCellTech, Salt Lake City, UT, United States of America; Children’s Hospital Boston, UNITED STATES

## Abstract

Globally, individuals with autoimmune Type 1 diabetes mellitus (T1DM) continue to depend for survival on insulin injections. While pancreas and intrahepatic pancreatic islet transplants can produce insulin-independence and ameliorate serious complications, both therapies depend on potentially toxic anti-rejection drugs. Furthermore, the scarcity of pancreas donors and islet transplant failures limit the general availability of such interventions. Recently, fetal and induced Pluripotent Stem Cells have been successfully differentiated to generate insulin producing β-like cells that generate euglycemia in diabetic mice. However, their clinical use still depends on anti-rejection drugs or immune-isolating encapsulation systems. We reported recently that allogeneic “Neo-Islets” (NI), 3-D organoids of Mesenchymal Stromal and Islet Cells are immune protected and permanently correct autoimmune diabetes in NOD mice by omental engraftment and endocrine cell redifferentiation. This new “endocrine pancreas” delivers islet hormones physiologically into the hepatic portal vein. Furthermore, treatment of insulin-dependent dogs with allogeneic canine NIs (ongoing FDA-approved Pilot Study) consistently improved glycemic control without the need for antirejection drugs. As there remains a critical need for curative therapies of T1DM, we engineered human NIs and tested their ability, after i.p. administration, to reestablish euglycemia in streptozotocin (STZ)-diabetic NOD/SCID mice. This diabetes model reproduces, in part, the clinical situation in which recipients of allogeneic biotherapies must take potent anti-rejection drugs that similarly create a life-long immune-compromised status. The present study demonstrates that human NI therapy (2x10e5/kg bw NIs/mouse) of STZ-diabetic NOD/SCID mice (n = 6), compared to controls (n = 6) significantly improved glycemic control, and most importantly, that a second dose given to the initial group normalized blood glucose levels long-term. Conclusion: Despite the limitations of the utilized diabetic NOD/SCID mouse model, the obtained data show that human NIs are curative, an observation that has high translational relevance and significantly supports the planned conduct of clinical trials with human NIs.

## Introduction

Therapeutic re-establishment of insulin independence in patients with T1DM that does not require potentially toxic antirejection drugs and a therapy that is globally available is urgently needed in order to improve the quality of life and prognosis of millions of insulin-dependent diabetic patients worldwide. Effective current treatments are allogeneic pancreas or islet transplants, both of which can provide endogenous insulin replacement and normalized glycemic control. However, the limited availability of donors, the need for repeated islet transplants, and the reliance on often toxic antirejection drugs limits the safety and use of such therapies [1, 2]. Because both allogeneic islet and pancreas transplants, and several experimental, allogeneic, insulin producing, stem- or precursor cell-based therapies still depend on the life-long use of potentially toxic antirejection drugs [3–6], auto- and allo-immune isolation of beta-like cells is being tested with various encapsulation technologies. However, these have frequently failed over time due to foreign body reactions, and some “open” capsule designs continue to require anti-rejection drugs [7–11]. The co-transplantation of immune-modulatory enhancing cells such as tolerogenic dendritic cells, autoantigen-specific Tregs, or autologous Hematopoietic Stem Cells along with insulin producing cell-based therapies has been proposed as a means of overcoming the need for encapsulation technologies, but such a cotransplantation strategy has yet to be tested [12].

We previously demonstrated that allogeneic “Neo-Islets” (NIs), three-dimensional organoids composed of approximately equal numbers of Mesenchymal Stromal Cells (MSCs) and culture expanded Islet Cells (ICs) do, when administered intraperitoneally, restore permanent euglycemia in diabetic NOD mice after spontaneous omental engraftment, endocrine cell redifferentiation, up regulation of Tregs and importantly without the use of encapsulation devices or antirejection drugs [13]. Similarly, when canine NIs (cNIs), composed of Adipose-derived Stromal or Stem Cells (ASCs) and cultured islet cells were administered i.p. to Streptozotocin-diabetic NOD/SCID mice, such mice were rendered euglycemic long term through exclusive secretion of canine insulin as demonstrated both by ELISA and subsequent removal of omentally engrafted cNIs [13, 14], resulting in prompt return of hyperglycemia. In order to test this promising biotherapeutic in a larger animal model and a more real-world setting, we are conducting an FDA-guided pilot study (INAD 012–776), treating spontaneously insulin-dependent pet dogs with allogeneic cNIs, using adapted protocols shown to be effective in NOD mice. Accordingly, canine NI (cNI)-treated dogs have shown a durable improvement in glycemic control, coupled with a reduction in the need for insulin, both achieved without the use of encapsulation devices or antirejection drugs [14]. So far, such effects have lasted greater than 3 years (unpublished observations from ongoing study), and without adverse events or allo-immune responses to NIs.

The positive results in our two, proof-of-concept studies conducted in two species strongly indicate that this therapy could be translated to the treatment of human Type 1 diabetes mellitus (T1DM). Accordingly, the present study was designed to test the ability of human NIs, 3-D organoids of human, bone marrow-derived MSCs and culture expanded human islet cells, aggregated in a 1:1 ratio, to produce durable euglycemia in Streptozotocin-diabetic NOD/SCID mice. This diabetes model resembles, to a significant extent, the clinical situation in which recipients of allogeneic pancreas or islet transplants must take potent anti-rejection drugs, which does also create a life-long immune-compromised status that exists in NOD/SCID mice. The present study demonstrates that human NIs administered i.p. to STZ-diabetic NOD/SCID mice, compared to controls, significantly improved glycemic control, and most importantly, that a second dose given to the initial group normalized blood glucose levels long-term. These observations show, despite obvious limitations of the utilized diabetic NOD/SCID mouse model, that human NIs are curative, an observations that has high translational relevance and significantly supports the planned conduct of clinical trials with human NIs.

## Materials and methods

### Reagents

Reagents used and their manufacturers are listed as indicated below, except for PCR reagents and primers that are listed in S1 Table.

### Study design

The current, preclinical study was undertaken in anticipation of a Phase 1 Clinical Trial with two objectives: to determine (a) whether human NIs (hNIs) can also restore euglycemia, and (b) whether redosing of suboptimally controlled diabetic animals could fully restore euglycemia in streptozotocin (STC)-diabetic Non-Obese Diabetic/Severe Combined Immunodeficiency mice (NOD/SCID, Harlan), as has been previously shown for mouse cell-derived NIs (mNIs), and to some extent for dog cell-derived NIs (cNIs) [13, 14]. Since these NIs are composed of human cells, and since human MSCs do not maintain immune evasive abilities in a xenogeneic setting (unpublished results), the NOD/SCID model was used. It does reproduce, in part, the clinical situation in which recipients of allogeneic biotherapies must permanently take potent anti-rejection drugs that similarly create a life-long immune-compromised status. Passaged hICs and hNIs that were to be used to treat diabetic NOD/SCID mice were characterized for gene expression profiles by rtPCR. For *in vivo* testing, NOD/SCID mice were made diabetic with STZ, then randomized based on blood glucose levels into groups of 6 each. Following randomization, the mice were administered insulin pellets (Linbits, Linshin Canada) to control blood glucose levels and prevent glucotoxicity [15–17] and enhance in vivo redifferentiation [11, 15, 18] of the graft. Once blood glucose levels were stabilized near normal, animals were treated i.p. either with ~2x10e5 human cell-derived NIs/kg bw (n = 6) or vehicle (n = 6), then followed for 8 weeks. Once placed in a group, and until endpoint or euthanasia for humane reasons, data from all animals were included in subsequent analyses. Six animals per group was chosen as that is the minimum number that would allow for valid statistical analysis and meaningful interpretation of results based on power analysis. Once blood glucose levels were determined to be no longer significantly improved compared to controls without administration of exogenous insulin, mice in each group were again treated with either 2x10e5 NIs/kg bw or vehicle, and followed for an additional 6 weeks.

### Animal model

Animal studies were conducted in adherence to the NIH Guide for the Care and Use of Laboratory Animals, and were supervised and approved by an institutional veterinarian and member of the IACUC.

#### Care

NOD/SCID mice were maintained in a sterile environment, and provided with sterile bedding, food and water. They were kept in a temperature and humidity controlled environment on a 12 hr light dark cycle and given free access to food and water. Animal health and behavior were visually observed at least once a day during the work week, and by blood glucose and weight checks at least 2 times a week by staff, each of whom had completed CITI training in the care of rodents, and had at least 2 years’ experience with mice and the procedures herein described.

#### Induction of diabetes and treatment

12 female, 13 week old NOD/SCID mice were made diabetic with one to two i.p. doses of Streptozotocin (STZ; Sigma), 200 mg ip dissolved in citrate buffer (pH 4.5; Sigma), and administered under light anesthesia as described below. Tail vein blood glucose levels were monitored 2x per week, and mice were considered diabetic when such levels were > 300 mg/dL for 2 consecutive days, at which point, mice were lightly anesthetized and treated with sub-cutaneous, slow-release insulin (Linbit) pellets. Once blood glucose levels were controlled to < 200 mg/dL, the two groups of six mice each were lightly anesthetized, and treated i.p. either with (i) 2x10e5 human cell derived NIs (hNIs/kg b.wt.; in 500 uL DMEM (5mM glucose) (Gibco)) or (ii) vehicle (500 uL DMEM (5mM glucose)).

#### Anesthesia

Mice were anesthetized with isoflurane (Baxter), 1–5%, using an inhalation rodent anesthesia system (Euthanex). Rectal temperatures were maintained at 37°C using a heated surgical waterbed (Euthanex).

#### Blood glucose and weight monitoring

Blood glucose concentrations were assessed twice per week via sterile tail vein sampling, using a 27–30 gauge needle to obtain a drop of blood, and a OneTouch Ultra 2 glucometer (level of detection, 20–600 mg glucose/dL; LifeScan). Post blood sampling, mice were observed until bleeding stopped and for a short time after for signs of tail bruising or pain (hunched appearance, head pressing, etc.). As anesthesia results in a rise of blood glucose, anesthesia was not used for blood glucose monitoring. Care was taken to minimize the pain and distress caused to mice required by handling and blood sampling for glucose monitoring, and analgesics were available as described in pain management below for any animal showing signs of pain from tail vein sampling. Animal weight was assessed twice weekly in conjunction with blood glucose monitoring.

#### Intraperitoneal Glucose Tolerance Tests (i.p. GTTs) and assay of human insulin

At indicated time-points post 1^st^ and 2^nd^ doses of hNIs, vehicle-treated and hNI-treated NOD/SCID mice, and an additional group of age-matched, non-diabetic NOD/SCID control mice (n = 6) were fasted 5 hrs, whereupon baseline blood glucose levels were measured. Animals were anesthetized and 2 g glucose/kg b.wt. (dissolved in 0.5 ml serum free medium and filter sterilized; Sigma, St. Louis, MO) were administered via i.p. injection. Tail vein blood glucose levels were determined at 30 min, 60 min and 120 min post glucose administration. Human insulin levels in the sera of hNI and vehicle treated groups of mice were assayed by ELISA, following the manufacturer’s instructions (Mercodia, Uppsala, Sweden).

#### Pain management

Buprenorphine 0.05 mg/kg bw IM was available as needed for any animal appearing to suffer from pain following i.p. STZ administration, NI administration, i.p. glucose tolerance testing, or tail vein sampling.

#### Endpoint criteria

For all mice in this study, the following criteria were used to determine whether they should be removed from the protocol or euthanized to prevent suffering: Animals that exhibited evidence of poor health, including weight loss greater than 20%, excessive wasting (>20% compared to age/sex matched littermates), ungroomed appearance, poor activity level, labored breathing or loss of appetite/water intake, neoplasia, stupor, severe injury due to fighting with cage mates, any signs of abnormal behavior including severe aggressiveness towards handler or cage mates such as to inflict injury, lack of physical or mental alertness, or any animal appearing to be in grave distress. Animals beginning to show signs of distress were monitored daily and carefully observed for general appearance, behavior and weight loss. Any animal appearing to be in grave distress or to have weight loss or muscle wasting of 20% or more were immediately euthanized to prevent further suffering.

No animal died before meeting endpoint criteria or study endpoint, but four mice in the vehicle treatment group met the criteria for euthanasia (all four exhibited excessive wasting and lack of appetite, combined with ungroomed appearance), and were euthanized on days 46 (3 mice) and 56 (1 mouse) as detailed in “Euthanasia” below, and as soon as they met those criteria.

#### Euthanasia

At study endpoint (applied to 8 of the 12 mice in the study), 15 weeks post first treatment with STZ, and where necessary as defined by endpoint criteria (applied to 4 mice in the vehicle treatment group) mice were euthanized using CO2 gas / 4–5 L over 2–4 minutes. Death was verified by the assurance of the cessation of respiratory and cardiovascular movements by observation for at least 10 minutes.

### Cells

NIs are composed of equal numbers of culture-expanded human MSCs and human Islet Cells, which spontaneously form clusters when co-cultured. Culture and NI formation are detailed below.

#### Islet cell culture

Research grade human islets from adult, non-diabetic donors were purchased from Prodo Labs. Islet cells were cultured by placing whole islets into tissue culture flasks and culturing them in RPMI 1640 (Life Technologies) + 10% human Platelet Lysate (hPL; Cell Therapy and Regenerative Medicine, Salt Lake City, UT) + Gentamycin, Penicillin, Streptomycin (GPS; Sigma) until 90% confluent. For passaging, cells were trypsinized using 1x Trypsin EDTA (Sigma), pelleted by centrifugation at 600x g for 5 min, washed with DMEM (5mM glucose) + 10% hPL + GPS, and reseeded at a density of 2x10e5 cells into Cell Bind coated T75 flasks (Corning). Cultured Islet Cells (IC) were used at Passage 1.

#### MSC culture

Human, bone marrow derived MSCs were purchased from Lonza (Walkersville, MD) and cultured as previously described [13, 19, 20]. MSCs were used at P3 for NI formation.

#### Neo-Islet (NI) formation

**Fig 1:**
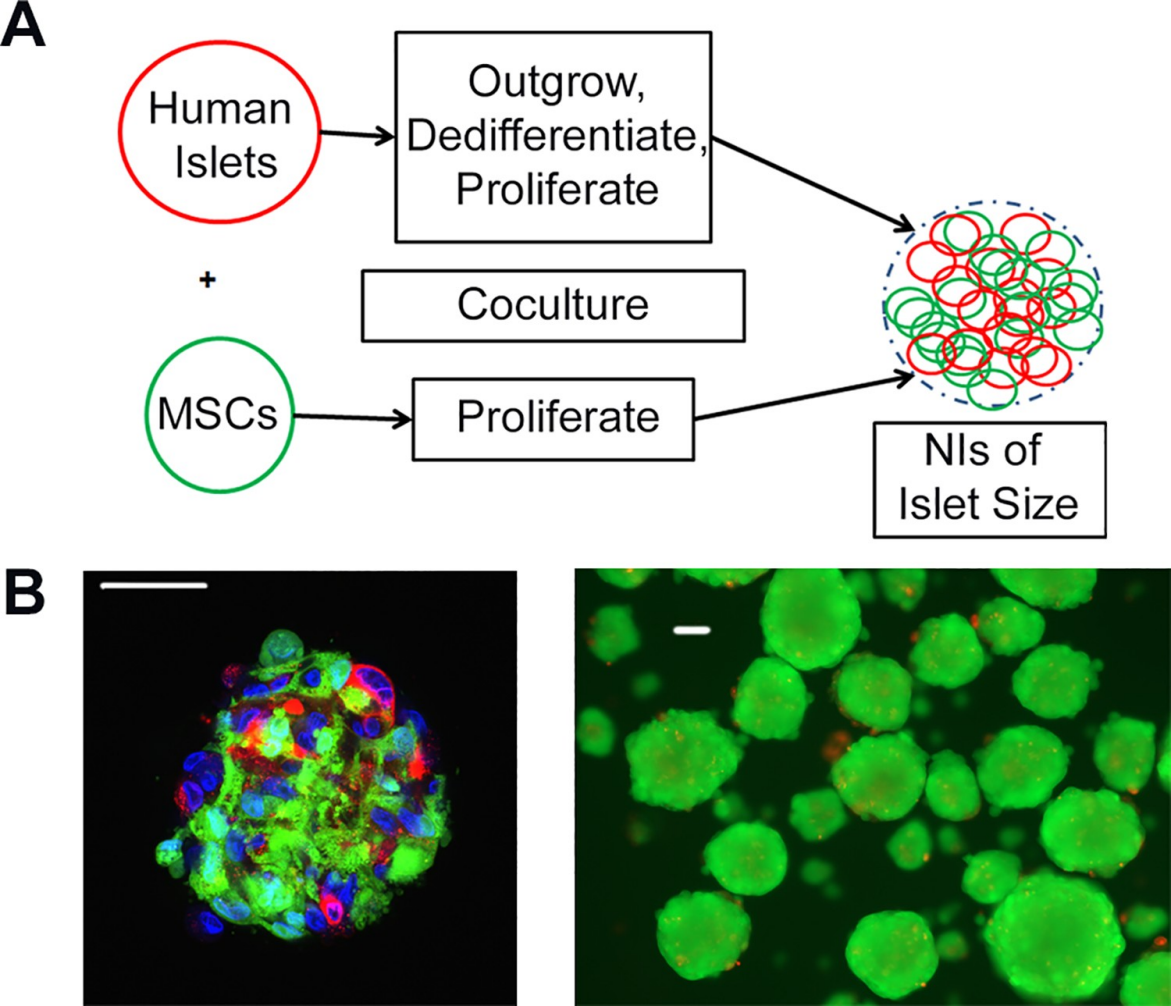
NI production. (A) Schematic of allogeneic human Neo-Islet production from human Islet Cells, outgrown from intact islets, and bone marrow-derived human Mesenchymal Stromal Cells (MSC). (B) Left: Confocal image of a single human Neo-Islet. Red: human Islet Cells. Green: human Mesenchymal Stromal Cells. Blue: Nuclei, DAPI stained. Scale bar 50 μm. Right: Aliquot of freshly prepared human Neo-Islets stained with PI/FDA for viability. Scale bar 50 μm.

MSCs and Islet cells were co-cultured in DMEM (5mM glucose) + 10% hPL at a 1:1 ratio in ultra-low adhesion surface culture dishes (Corning), and NIs formed overnight as previously described [13, 14] and as shown in Fig 1.

### rtPCR

Prior to *in vivo* administration, NIs were tested by rtPCR for expression of islet- associated genes INS, GCG, SST, PPY, PDX1, and UCN3. rtPCR was carried out as previously described [13], using the reagents and primers listed in the S1 Table. In brief, Relative Quantification, (RQ; defined as is standard as 2-ΔΔCT where CT is the Cycle Threshold [21]), was calculated through normalization to internal (deltaCT; beta actin and beta 2 microglobulin) and external controls (delta-deltaCT; parent cells), both accomplished using the ABS 7500 Real Time PCR System and software. Results are presented as log10(RQ) ± log10(RQmin and RQmax) so that up- and down-gene regulation is represented equally [22]. Differences between expression levels greater than log10(RQ) 2 or log10(RQ) -2 were considered significant [22].

### Statistical analysis

Data are expressed as Mean ± SEM or Mean ± 95% confidence interval, as indicated. Primary data were collected using Excel (Microsoft, Redmond, WA), and statistical analyses were carried our using Prism (GraphPad). Two tailed t-tests were used to assess differences between data means. A *P* value of < 0.05 was considered significant. For rtPCR, data are presented as Log10RQ, and statistical significance is defined as ≥ ± 2, as described in [22].

## Results

### Reduced levels of islet-associated genes are expressed in hNIs

hNIs were formed, each set incorporating P3 hMSCs and P1 islet cells from non-diabetic, adult human donors. hMSCs were obtained pre-characterized for expression of MSC-specific epitopes and genes, and for their ability to undergo trilineage differentiation (adipo-, osteo- and chondrogenic) (Lonza).

**Fig 2:**
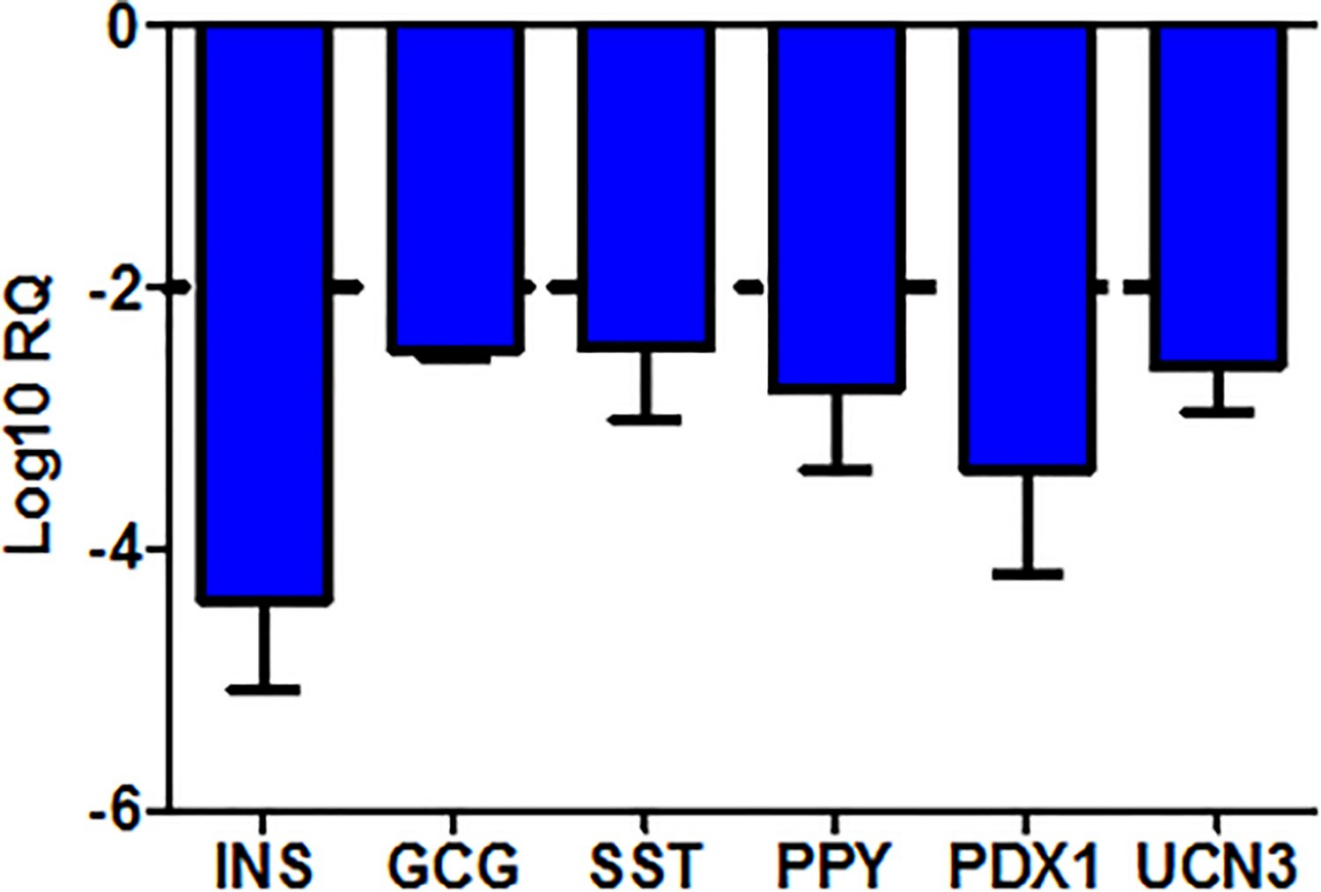
NI gene expression profiles. Shown are gene expression profiles of freshly prepared human Neo-Islets (hNIs) prior to i.p. administration to diabetic NOD/SCID mice, normalized to expression levels of whole, uncultured human islets. Log10 (RQ) values were calculated for NIs and graphed as the mean ± SEM. Log10(RQ) ≥ ± 2 (hashed line) was considered statistically significant. Islet associated genes (INS, GCG, SST, PPY, PDX1, and UCN3) are expressed in human NIs prior to administration, but at significantly reduced levels compared to those of freshly isolated human islets.

Gene expression analysis was conducted using rtPCR on the freshly formed NIs to determine their expression levels of islet endocrine genes as compared to those of whole islets. As expected from previous experiments using mouse and dog cells [13, 14], and as shown in Fig 2, NIs containing P1 islet cells expressed INS, GCG, SST, PPY, PDX1, and UCN3 mRNAs. Also as was previously found for mouse and dog cells, respective expression levels of these islet cell genes were significantly reduced compared to freshly isolated human islets. In other words, islet associated genes (INS, GCG, SST, PPY, PDX1, and UCN3) are expressed in human NIs prior to administration, but at significantly reduced levels compared to those of freshly isolated human islets, comparable to what was previously found for dog and mouse culture-expanded islet cells [13, 14].

### Therapeutic efficacy of hNIs

#### A single dose of hNIs improves glycemic control in diabetic mice

In order to assess the therapeutic efficacy of hNIs for the treatment of insulin-dependent DM, Diabetes was established in 12 female NOD/SCID mice, after which they were randomized into 2 groups of 6 mice each, and their blood glucose levels were controlled with slow-release insulin pellets (Linbits). Once blood glucose levels were controlled, mice were treated either with vehicle or hNIs as described in Methods. After this treatment, mice were followed for 8 weeks, at which time, an i.p GTT was conducted as described in Methods, in conjunction with an ELISA assay to detect the presence of human Insulin.

**Fig 3:**
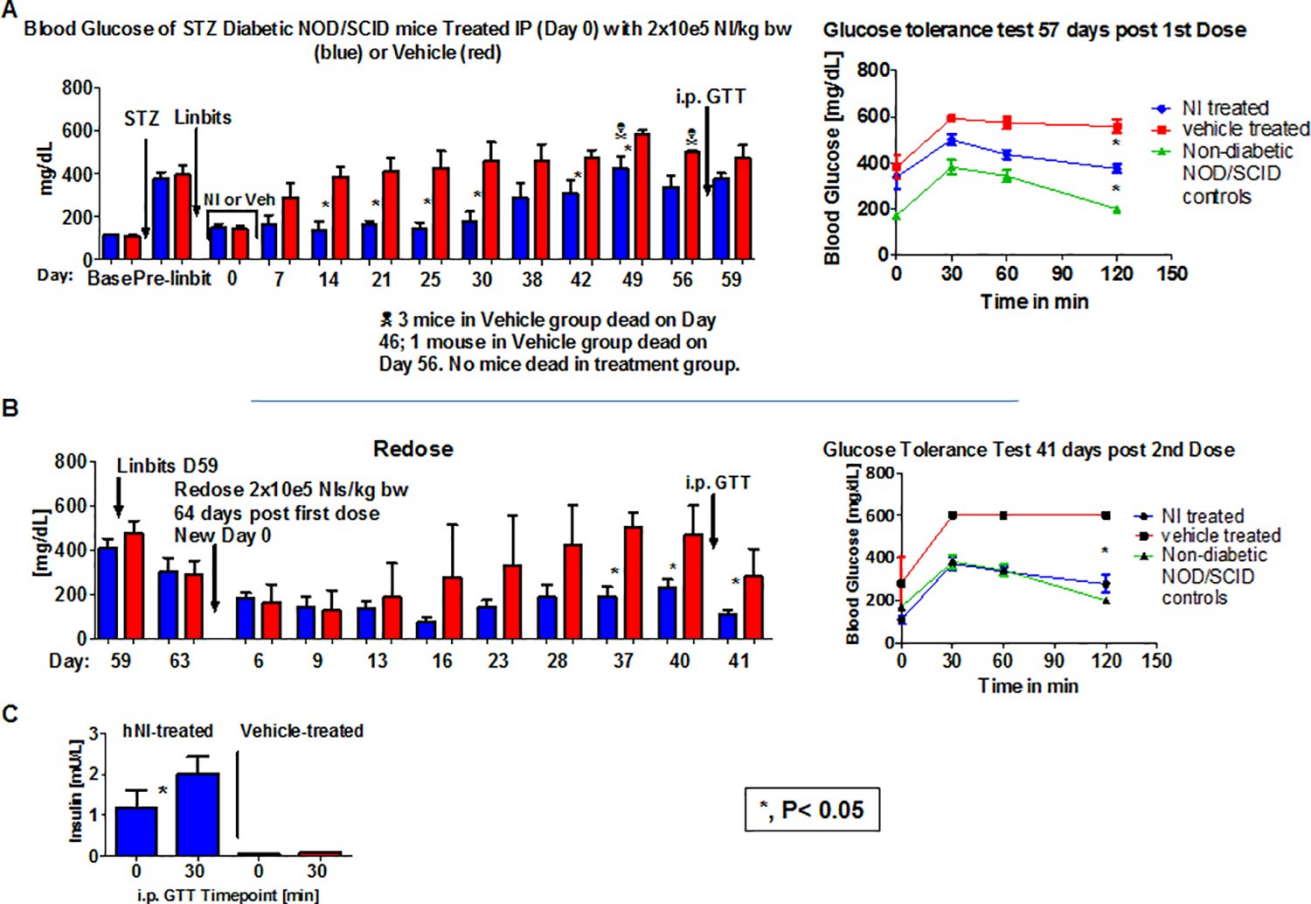
Therapeutic efficacy of single and repeat dosing of hNIs administered to diabetic NOD/SCID mice. (A) Human Neo-Islets given i.p. only transiently improve blood glucose levels and the i.p. Glucose Tolerance Test (right). (B) Upon i.p. redosing with the same number of hNIs blood glucose levels, and i.p. Glucose Tolerance Tests (ip GTT; right) are normalized compared to those in non-diabetic NOD/SCID mice, (C) a response mediated by the exclusive secretion of human Insulin in hNI re-treated NOD/SCID mice. As previously reported [12], murine insulin secretion during the ip GTT in non-diabetic NOD/SCID was physiological (not shown).

As shown in Fig 3A, administration of a single dose of hNIs to diabetic NOD/SCID mice improved glycemic control, as assessed by serum glucose measurements and i.p. GTTs, and this improvement is mediated by the exclusive secretion of human Insulin.

#### Administration of a second dose of hNIs establishes euglycemia in previously treated dysglycemic mice

While no mice in the treated group died, 4 animals died in the vehicle treated control group, and while hNI therapy significantly improved glycemic control vs. the vehicle group for 7 weeks, normoglycemia was not maintained (Fig 3A).

To test whether a second dose of hNIs could achieve euglycemia in the incompletely controlled mice shown in Fig 3A, at day 59 after the first dose, all surviving mice (n = 6 in the treatment group; n = 2 in the vehicle group) were again treated with insulin pellets (Linbits), and once blood glucose levels were normalized, they were re-dosed i.p. as before with 2x10e5 hNIs per kg bw (blue) or vehicle (red) on day 64 post the initial treatment. A second i.p. GTT was administered on day 41 post the second dose of hNI. After the second dose, blood glucose levels and i.p. GTTs (Fig 3B) were normalized in the hNI treatment group to the pattern observed in non-diabetic NOD/SCID mice, while the i.p. GTT in the vehicle treated group remained abnormal.

#### Human insulin is detected in serum from hNI- but not vehicle-treated mice

Serum collected during the i.p. GTT depicted in Fig 3B was assayed by ELISA for the presence of human insulin as described in Methods. Only serum from hNI treated, but not vehicle treated mice contained human insulin (Fig 3C). As previously reported [12], murine insulin secretion during the ip GTT in non-diabetic NOD/SCID was physiological (not shown).

## Discussion

Results presented herein indicate that as we have previously shown with mouse and dog NIs, human cell derived NIs are effective in reducing or eliminating the need for insulin in diabetic animals. Importantly, redosing is effective in completely eliminating the need for insulin in partly controlled, previously NI treated animals.

These results parallel those previously observed wherein dog cell derived NIs were used to render STZ diabetic NOD/SCID mice euglycemic [13]. The fact that this analogous human product has a similar gene expression profile to that of the dog and mouse product further suggests this therapy holds promise for successful translation to the clinic [13, 14].

The above described preclinical work using dog NIs to treat diabetic NOD/SCID mice xenogeneically led to the ongoing pilot study in which canine NIs are being used allogeneically to treat spontaneously diabetic pet dogs. These treated dogs have shown stable, durable (3 years) and statistically significant reduction in their needs for insulin, as well as significantly improved glycemic control, without the need for anti-rejection drugs or the development of significant adverse events [14]. The here presented preclinical study, along with the previous translation of this technology to the veterinary setting further justifies our plans to pursue an IND for the conduct of a clinical trial with allogeneic human NIs. Of particular importance is the demonstration that animals that showed incomplete glycemic control with the first dose of hNIs achieved durable euglycemia after receiving a second, identical dose of hNIs.

Human islet transplants, the most closely analogous cellular therapeutic to the here presented NI therapy, are generally administered in more than one dose to be fully effective. It has been observed that if islet transplants are administered in only one dose, the need for insulin will frequently rebound [23]. Although with dog cell-derived NIs given to diabetic pet dogs we have not seen this rebound in the need for insulin, we have to date only observed a reduction in the need for insulin, and not an elimination of that need [14]. The situation with Neo-Islet therapy may be similar to that of human islet transplants in the requirement for a second dose in order to be “curative” in dogs and humans. Thus, the observed ability to further improve glycemic control of diabetic mice through redosing is significant in its implications for therapeutic dosing strategies in that doses of NIs can be adjusted either in NI number or frequency, and if necessary, a second dose can be safely administered. This would likely be effective both in dogs and in humans in further reducing or eliminating the need for insulin, and is likely safe given that, as previously shown [13, 14], the first dose of allogeneic NIs is not rejected, and no immune response to administered cells has been observed to date in allogeneically NI treated mice or dogs [13, 14]. Further supporting the above conclusions, one dog has been redosed with thus far positive results and no immune response to the second dose of allogeneic NIs has been seen (unpublished observation).

NI therapy holds promise in that it overcomes the principal technical hurdles that thus far have limited the use and success of cell based therapies for autoimmune mediated T1DM, specifically, the need for antirejection drugs or encapsulation devices; the difficulty of expanding beta cells in culture; and the shortage of pancreas donors [1, 24]. Culture expansion of islet cells as is done with the NI technology allows for the production of many therapeutic doses from a single organ donor [25].

In conclusion, the present work demonstrates for the first time that human NIs, administered twice to STZ-diabetic NOD/SCID mice, functionally and durably cure their diabetic state. These observations are paralleled by the previous demonstration that canine NIs administered to STZ-diabetic NOD/SCID mice induce normoglycemia, an observation that is being successfully tested in an ongoing pilot study of insulin-dependent dogs [13]. The translational significance and utility of the employed NOD/SCID model, despite its obvious limitations, lies in the fact that patients who receive an allogeneic pancreas or islet or other cell-based transplant require the life-long administration of potent anti-rejection drugs that have potentially significant side effects related to induction of a permanent immune compromised state that resembles to a certain extent the severe, combined immune deficiency of NOD/SCID mice. Furthermore, the MSC component of NIs achieves and maintains immune isolation within the microenvironment of the transplanted organoids that prevents their rejection, i.e., there is no systemic immune compromise, which represents a distinct therapeutic and safety advantage. Finally, the NI technology overcomes the substantial donor scarcity by facilitating the production of high numbers of therapeutic doses from a single donor, an efficiency that is not accomplished in the field of islet transplantation. Taken together, we posit that the developed [12, 13] and here reported NI technology has reached sufficient scientific maturity that justifies the planned conduct of clinical trials in patients with T1DM.

## Supporting information

S1 Table PCR reagents used and their sources. (DOCX)

S1 File Data sets for figures. (PPTX)

S2 File (XLSX)
