# Supplementary material for: Intraperitoneal administration of human “Neo-Islets”, 3-D organoids of mesenchymal stromal and pancreatic islet cells, normalizes blood glucose levels in streptozotocin-diabetic NOD/SCID mice: Significance for clinical trials
Source: PLoS One. 2021 Oct 28;16(10):e0259043. doi: 10.1371/journal.pone.0259043 (PMC8553138; doi:10.1371/journal.pone.0259043)
Supplement: S1 Table — (DOCX) [file pone.0259043.s001.docx]

**S1 Table.** PCR Reagents used and their sources.

| **Reagent** | **Supplier** | **Catalog #** |
| --- | --- | --- |
| Rneasy Mini Kit | Quiagen | 74107 |
| SuperScript II reverse transcriptase | ThermoFisher Scientific | 18064014 |
| Random Primers | ThermoFisher Scientific | 48190011 |
| 100mM dNTP Set | ThermoFisher Scientific | 10297018 |
| TaqMan Universal Master Mix II with UNG | ThermoFisher Scientific | 4440038 |
| Water Nuclease-free | Invitrogen | AM9937 |
| PCR Plate | Life Technologies | 4306737 |
| Adhesive film | ThermoFisher Scientific | 4311971 |
|  |  |  |
| **PCR Primer for Gene** | **Vendor** | **Catalog #** |
| Beta Actin (ACTB) | Life Technologies | Hs01060665_g1 |
| Beta 2 Microglobulin (B2M) | Life Technologies | Hs00984230_m1 |
| Insulin (INS) | Life Technologies | Hs02741908_m1 |
| Glucagon (GCG) | Life Technologies | Hs01031536_m1 |
| Somatostatin (SST) | Life Technologies | Hs00356144_m1 |
| Pancreatic Polypeptide (PPY) | Life Technologies | Hs00358111_g1 |
| Pancreatic and Duodenal Homeobox 1 (PDX1) | Life Technologies | Hs00236830_m1 |
| Urocortin-3 (UCN3) | Life Technologies | Hs00846499_s1 |
