# Supplementary material for: Intraperitoneal administration of human “Neo-Islets”, 3-D organoids of mesenchymal stromal and pancreatic islet cells, normalizes blood glucose levels in streptozotocin-diabetic NOD/SCID mice: Significance for clinical trials
Source: PLoS One. 2021 Oct 28;16(10):e0259043. doi: 10.1371/journal.pone.0259043 (PMC8553138; doi:10.1371/journal.pone.0259043)
Supplement: S1 File — (PPTX) [file pone.0259043.s002.pptx]

## Slide 1
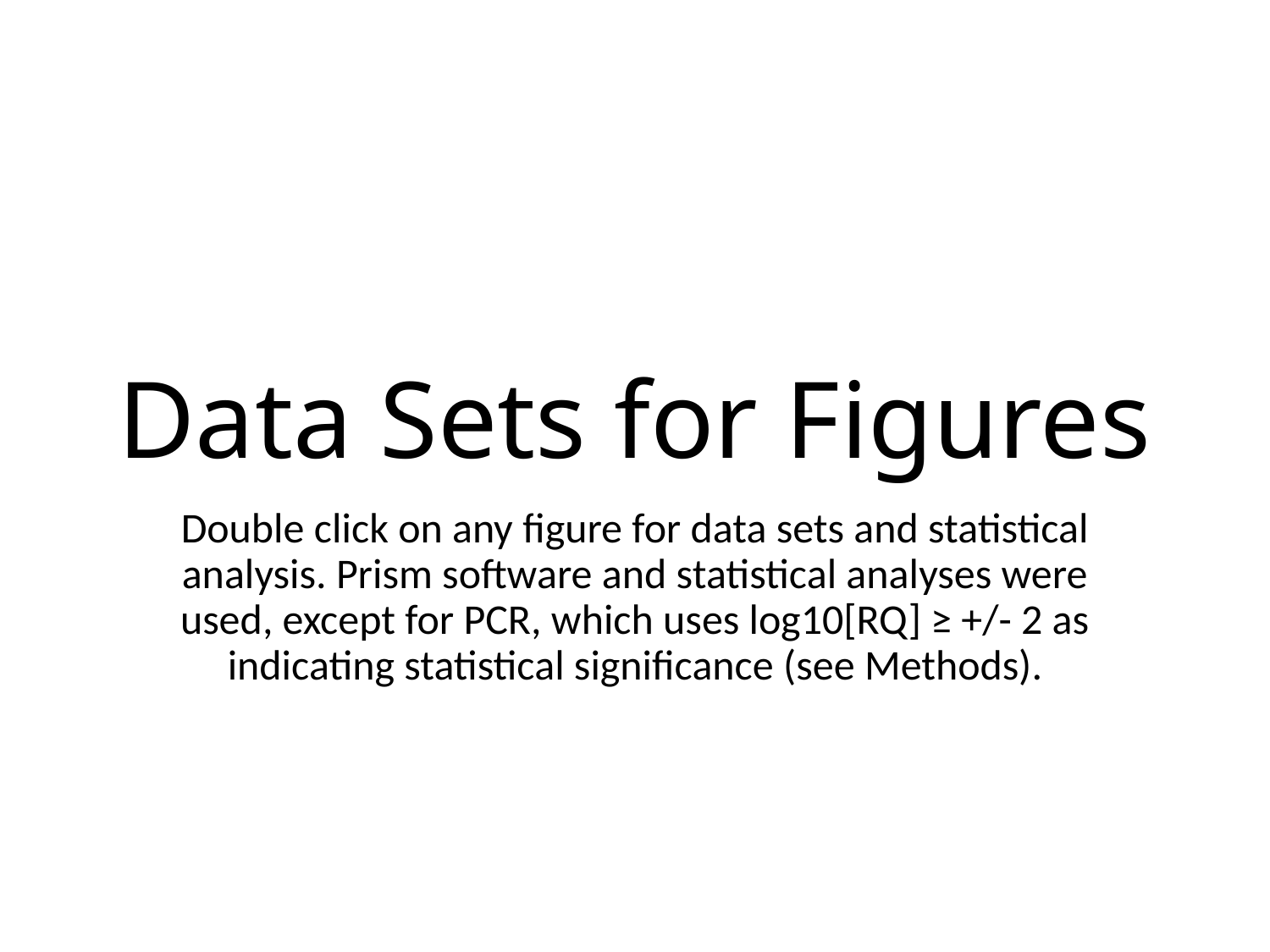

# Data Sets for Figures
Double click on any figure for data sets and statistical analysis. Prism software and statistical analyses were used, except for PCR, which uses log10[RQ] ≥ +/- 2 as indicating statistical significance (see Methods).

## Slide 2
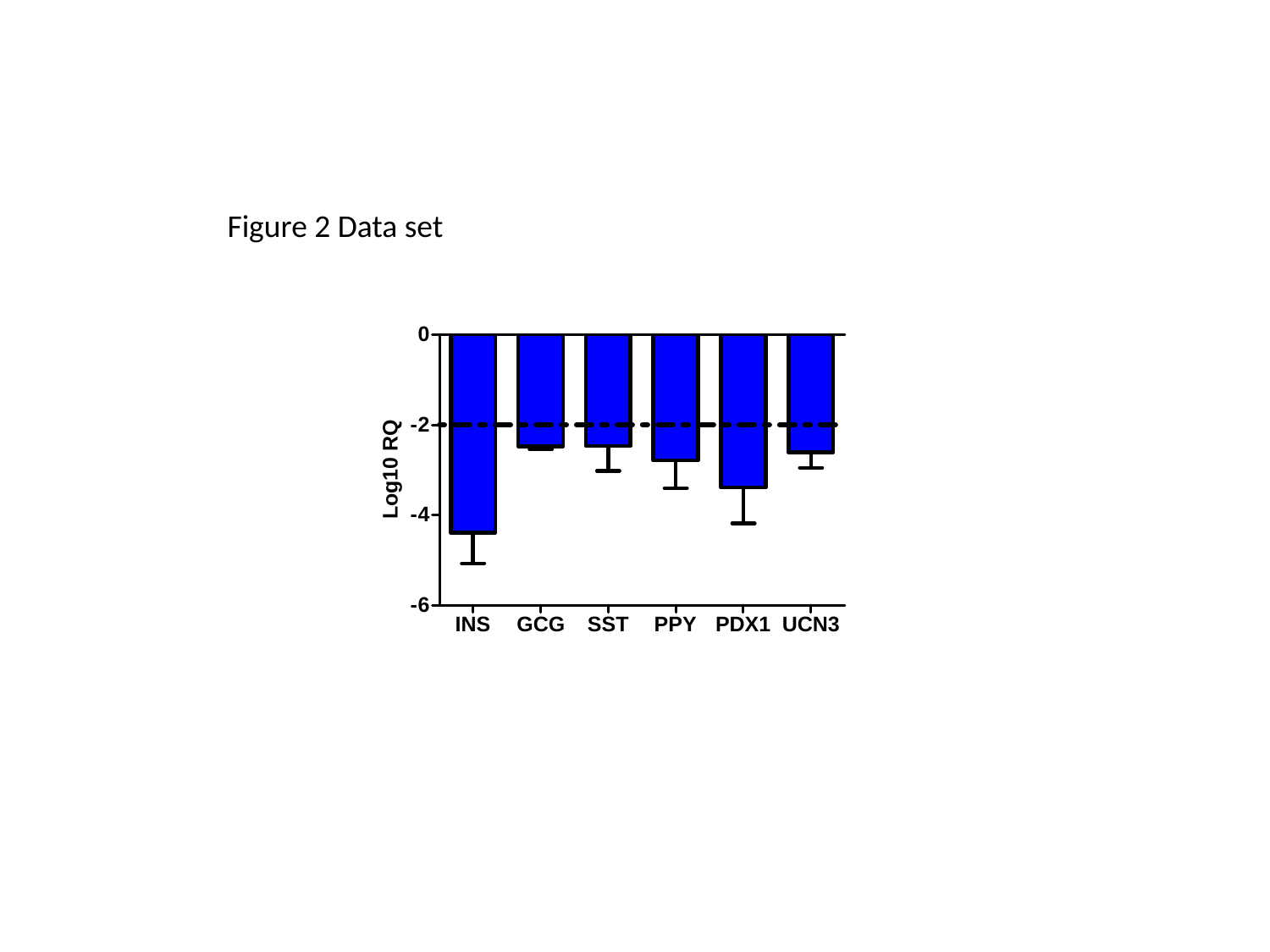

Figure 2 Data set

## Slide 3
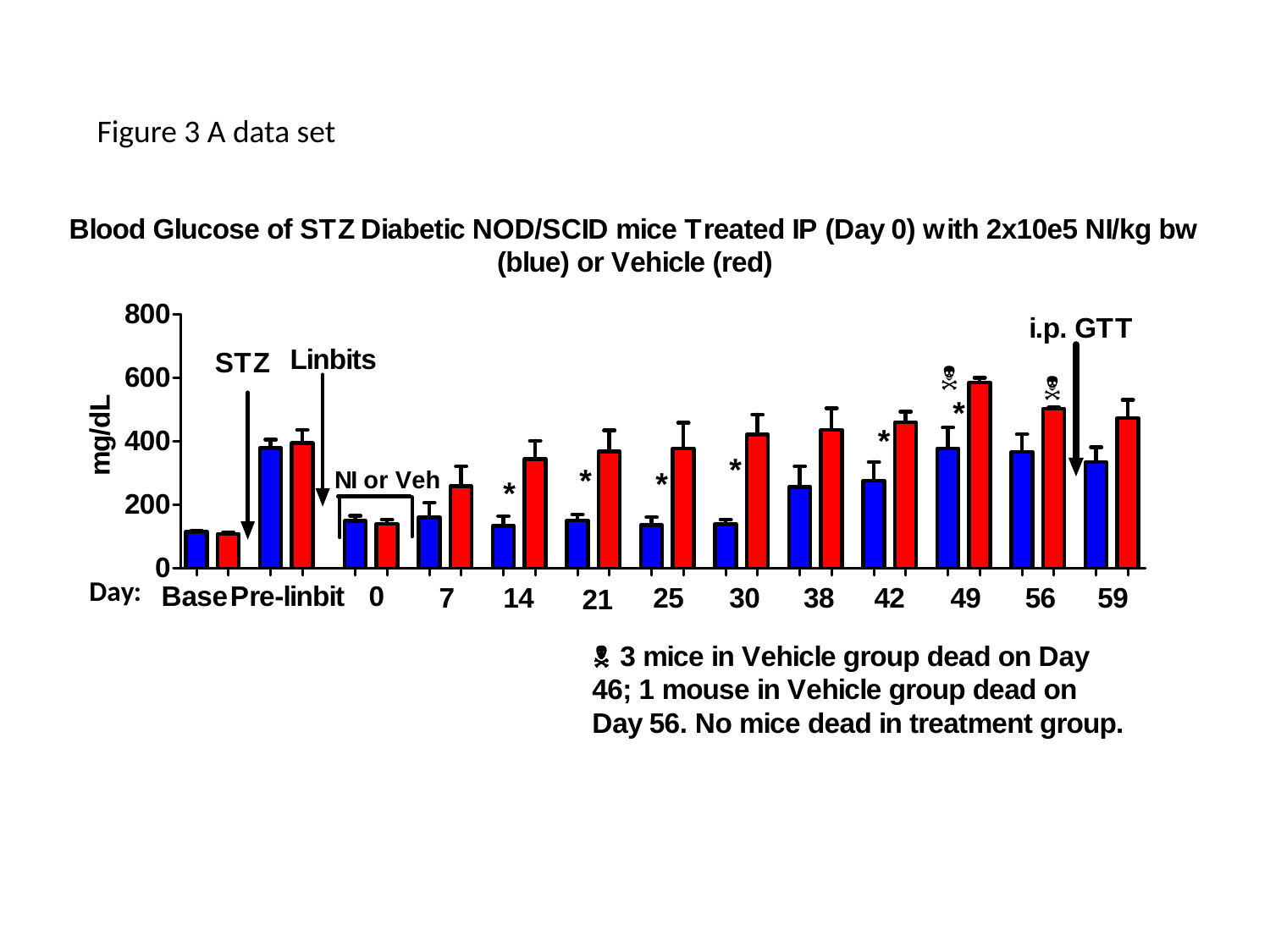

Figure 3 A data set

## Slide 4
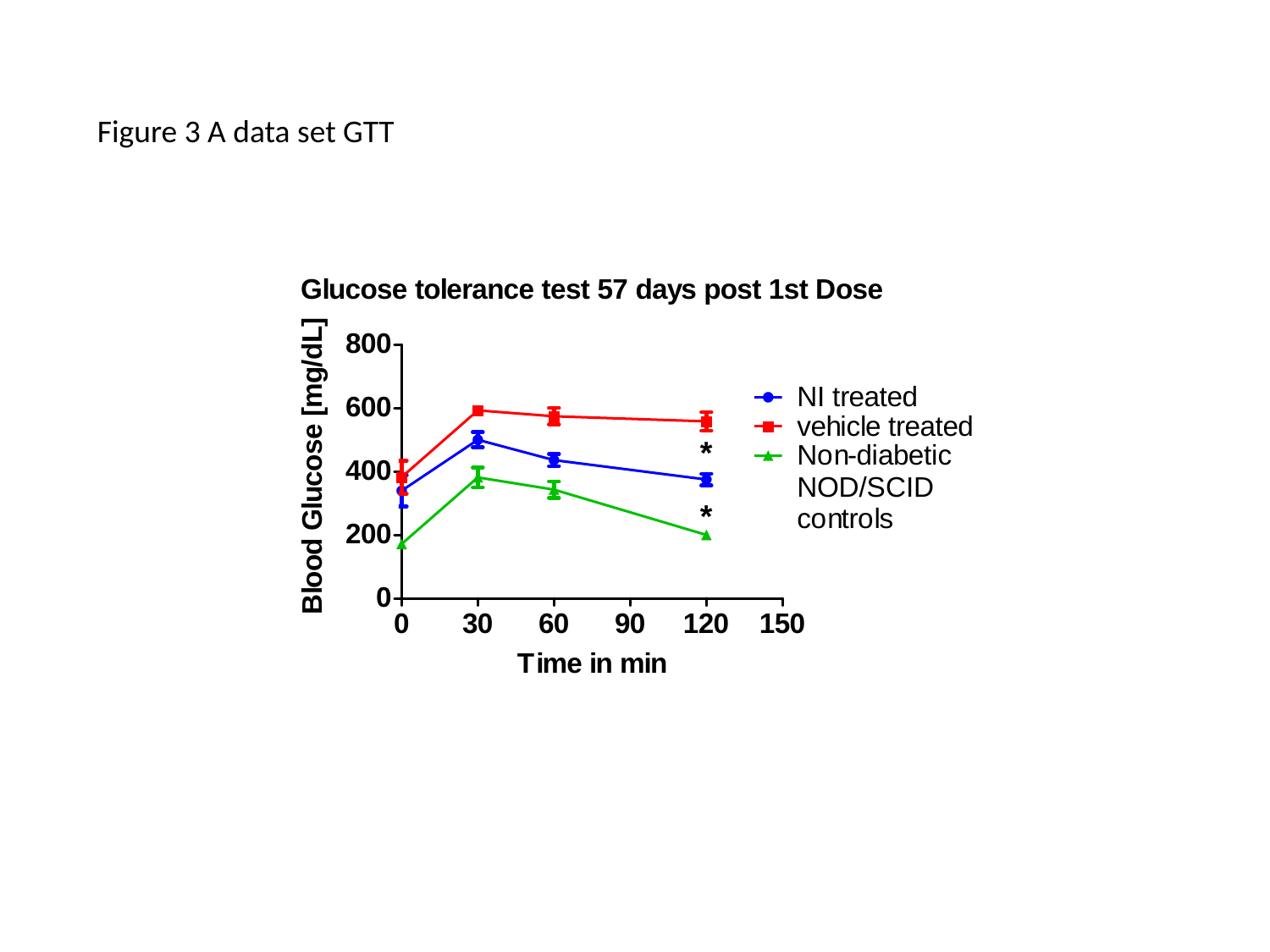

Figure 3 A data set GTT

## Slide 5
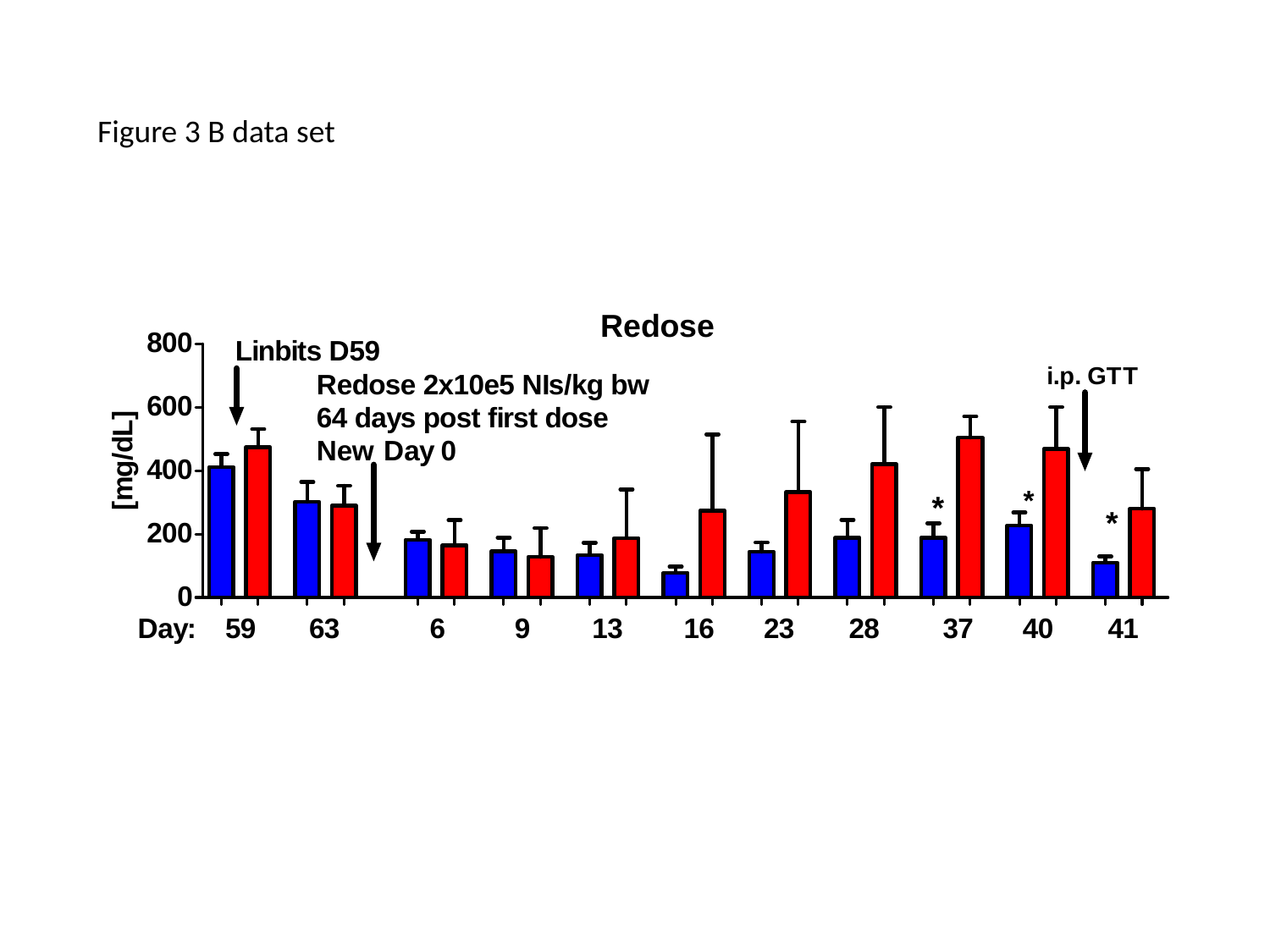

Figure 3 B data set

## Slide 6
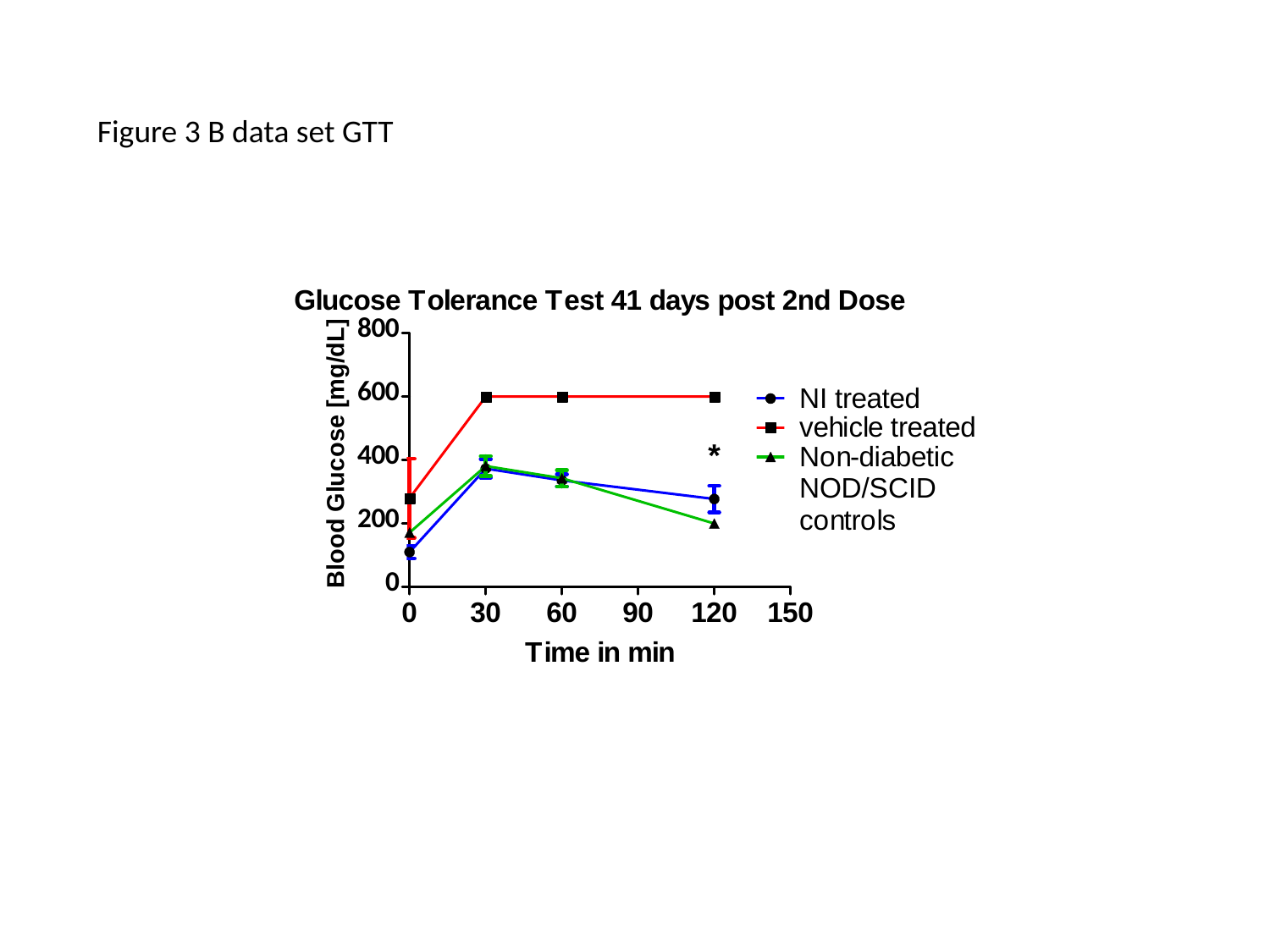

Figure 3 B data set GTT

## Slide 7
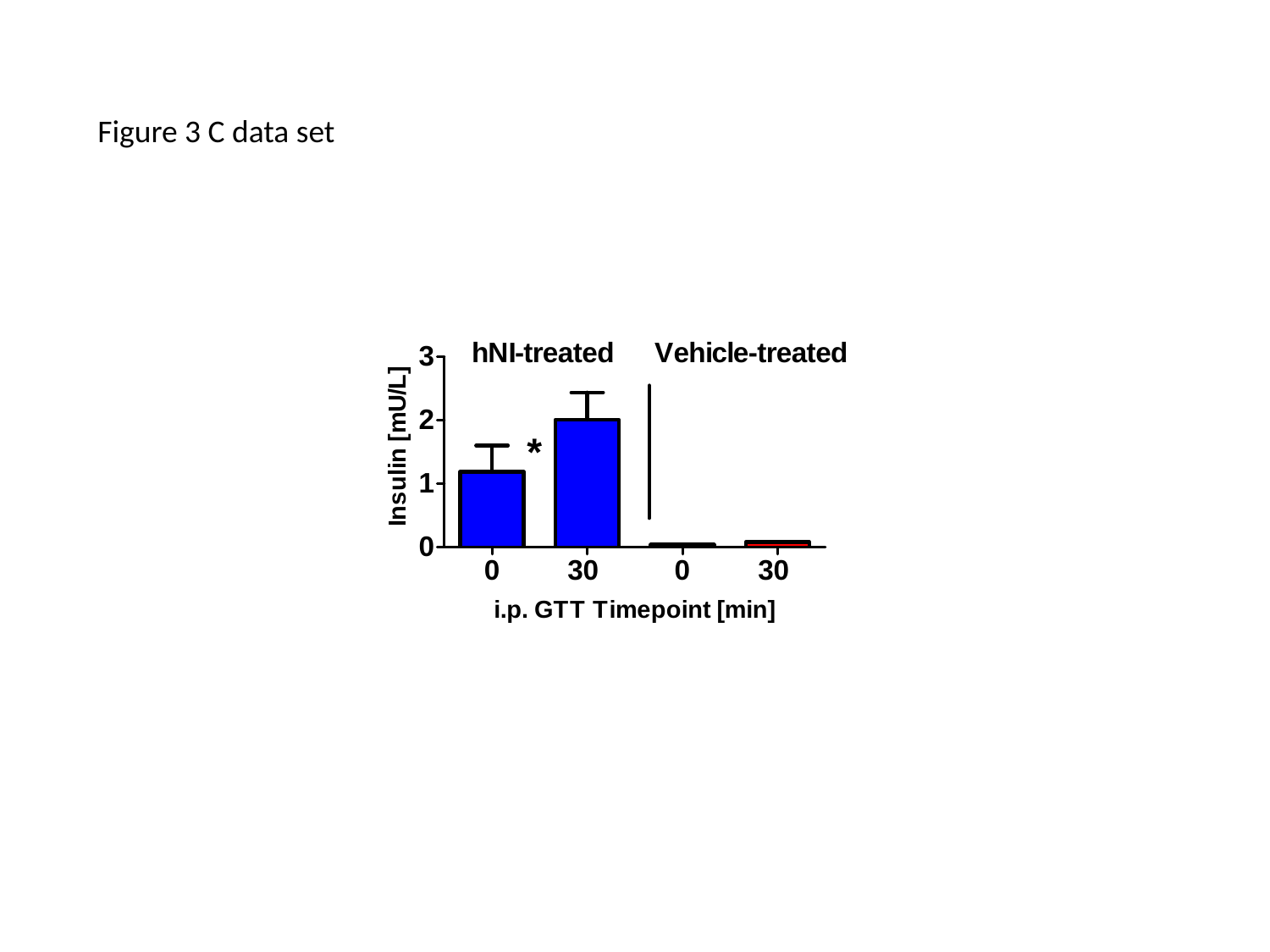

Figure 3 C data set
